# Automated classification of urine biomarkers to diagnose pancreatic cancer using 1-D convolutional neural networks

**DOI:** 10.1186/s13036-023-00340-0

**Published:** 2023-04-17

**Authors:** Mohamed Esmail Karar, Nawal El-Fishawy, Marwa Radad

**Affiliations:** 1 Department of Industrial Electronics and Control Engineering, Faculty of Electronic Engineering, Menoufia University, Al Minufiyah, Egypt; 2 Department of Computer Science and Engineering, Faculty of Electronic Engineering, Menoufia University, Al Minufiyah, Egypt

**Keywords:** Microfluidics, Pancreatic cancer, Urine biomarkers, Artificial intelligence, Convolutional neural networks, Long short-term memory

## Abstract

**Background:**

Early diagnosis of Pancreatic Ductal Adenocarcinoma (PDAC) is the main key to surviving cancer patients. Urine proteomic biomarkers which are creatinine, LYVE1, REG1B, and TFF1 present a promising non-invasive and inexpensive diagnostic method of the PDAC. Recent utilization of both microfluidics technology and artificial intelligence techniques enables accurate detection and analysis of these biomarkers. This paper proposes a new deep-learning model to identify urine biomarkers for the automated diagnosis of pancreatic cancers. The proposed model is composed of one-dimensional convolutional neural networks (1D-CNNs) and long short-term memory (LSTM). It can categorize patients into healthy pancreas, benign hepatobiliary disease, and PDAC cases automatically.

**Results:**

Experiments and evaluations have been successfully done on a public dataset of 590 urine samples of three classes, which are 183 healthy pancreas samples, 208 benign hepatobiliary disease samples, and 199 PDAC samples. The results demonstrated that our proposed 1-D CNN + LSTM model achieved the best accuracy score of 97% and the area under curve (AUC) of 98% versus the state-of-the-art models to diagnose pancreatic cancers using urine biomarkers.

**Conclusion:**

A new efficient 1D CNN-LSTM model has been successfully developed for early PDAC diagnosis using four proteomic urine biomarkers of creatinine, LYVE1, REG1B, and TFF1. This developed model showed superior performance on other machine learning classifiers in previous studies. The main prospect of this study is the laboratory realization of our proposed deep classifier on urinary biomarker panels for assisting diagnostic procedures of pancreatic cancer patients.

## Introduction

Pancreatic cancer (PC) is the third leading cause of death in the world as reported by cancer statistics in 2022 [1]. Pancreatic ductal adenocarcinoma (PDAC) is the most common type of exocrine tumor affecting the pancreas [2]. Although PDAC is the 12th most common cancer worldwide, its aggressive nature and the lack of obvious symptoms make it a major public health burden. The PDAC has the lowest 5-year overall survival rate of any malignancy due to late diagnosis (11%) [1]. The procedure of early PDAC diagnosis is the main key to surviving cancer patients. That requires a concerted effort among clinicians, radiologists, biologists, and computer scientists.

Clinical data is the initial stage in the diagnosing process of any disease. Electronic health records (EHR) represent tremendous heterogeneous data. EHRs contain clinical information such as diagnoses, procedures, information within clinical notes, and medications. Recent studies succeeded in identifying high-risk PDAC patients from national EHRs [3]. Such population-based studies improve awareness of PDAC risk and recommend patients for more diagnostic procedures like biomarker testing and medical image scanning [4]. Medical imaging-guided procedures are fundamental techniques for diagnosing PDAC, including magnetic resonance imaging (MRI), computed tomography (CT), endoscopic ultrasound (EUS), and Immuno-Positron Emission Tomography (Immuno-PET) [5]. Despite the difficulty of imaging early pancreatic cancer, numerous promising recent studies are reported in [6]. The high cost of radiological imaging makes it an unlikely choice for general PDAC screening. As a result, the researchers’ attention turns to utilizing biomarkers as a preliminary step toward PDAC early detection. There are rapid developments in genomic sequencing and their different strategies such as proteomics, epigenomics, and transcriptomics create large-scale multi-omics data. The Cancer Genome Atlas (TCGA) project [7] was established by the National Cancer Institute in 2006. It provides multi-omics data for more than 20,000 tumors spanning 33 cancer types. Many recent efforts have been made to integrate omics science with cancer research for different cancer types including PDAC [8, 9]. According to these studies, informative biomarkers with genomics can assist pathologists to get more advanced PDAC indicators.

Body fluids are rich with informative biomarkers that are crucial for the early identification of PDAC [10, 11]. For example, cyst fluid, pancreatic juice, and bile need invasive procedures like surgery or endoscopy to be collected. Blood is also a minimally invasive, inexpensive, and reproducible source of tumor biomarkers [12]. It is enriched with proteomic biomarkers such as carbohydrate antigen 19–9 (CA19-9) and transcriptomic biomarkers based on RNA sequencing which is called Circulating micro RNAs (miRNAs) [13, 14]. In addition, blood exosomes which are nano-sized, extracellular vesicles that carry various pathogenic RNAs, DNAs, and proteins were used to diagnose cancerous cells in the pancreas [15].

Urine represents a promising alternative body fluid for biomarker discovery. It is an ideal fluid for public diagnostic screening tests because patients may easily provide a significant volume of it in an entirely non-invasive inexpensive way [16]. Like blood, urine contains proteomic biomarkers in addition to transcriptomic biomarkers miRNAs. In 2015, Radon et al*.* [17] proposed a three-protein biomarker panel that is able to detect patients with early-stage PDAC in urine samples. They considered TFF1, LYVE-1, and REG1A as candidate proteomic biomarkers. On the micro-scale, a study reported the use of miRNA in urine for early detection of PDAC [18]. In 2020, Debernardi et al*.* [19] improved the existing panel by substituting REG1A with REG1B. In addition, they can differentiate between benign hepatobiliary disease and PDAC cases which represent a challenge in early-stage of PDAC because of the overlapping symptoms. They validate their panel using the PancRISK score [19]. The accurate detection and quantification of biomarkers in liquid biopsy are the millstone for the success of body fluid-based diagnostics methods which can be achieved using micro-and nano-based technologies [20].

Rapid technical innovation in microfluidics and nanofluidic technologies allows the detection of high-quality biomarkers from liquid biopsies with high specificity, and sensitivity [21, 22]. Different microfluidic chips have been designed for different body fluids such as blood [23], and urine [24]. Microfluidics technologies can improve cancer diagnosis by analyzing various tumor biomarkers such as circulating tumor DNA (ctDNA), circulating tumor cells (CTC), cell-free DNA (cfDNA), cell-free RNAs (cfRNAs), tumor-secreted exosomes, and proteins [25, 26]. However, the clinical interpretation of these biomarkers and their inter-relationships remain a challenge. Therefore, artificial intelligence (AI) plays an important role in assisting clinicians to automatically analyze the extracted biomarkers and detect PDAC at early stages.

**Fig. 1:**
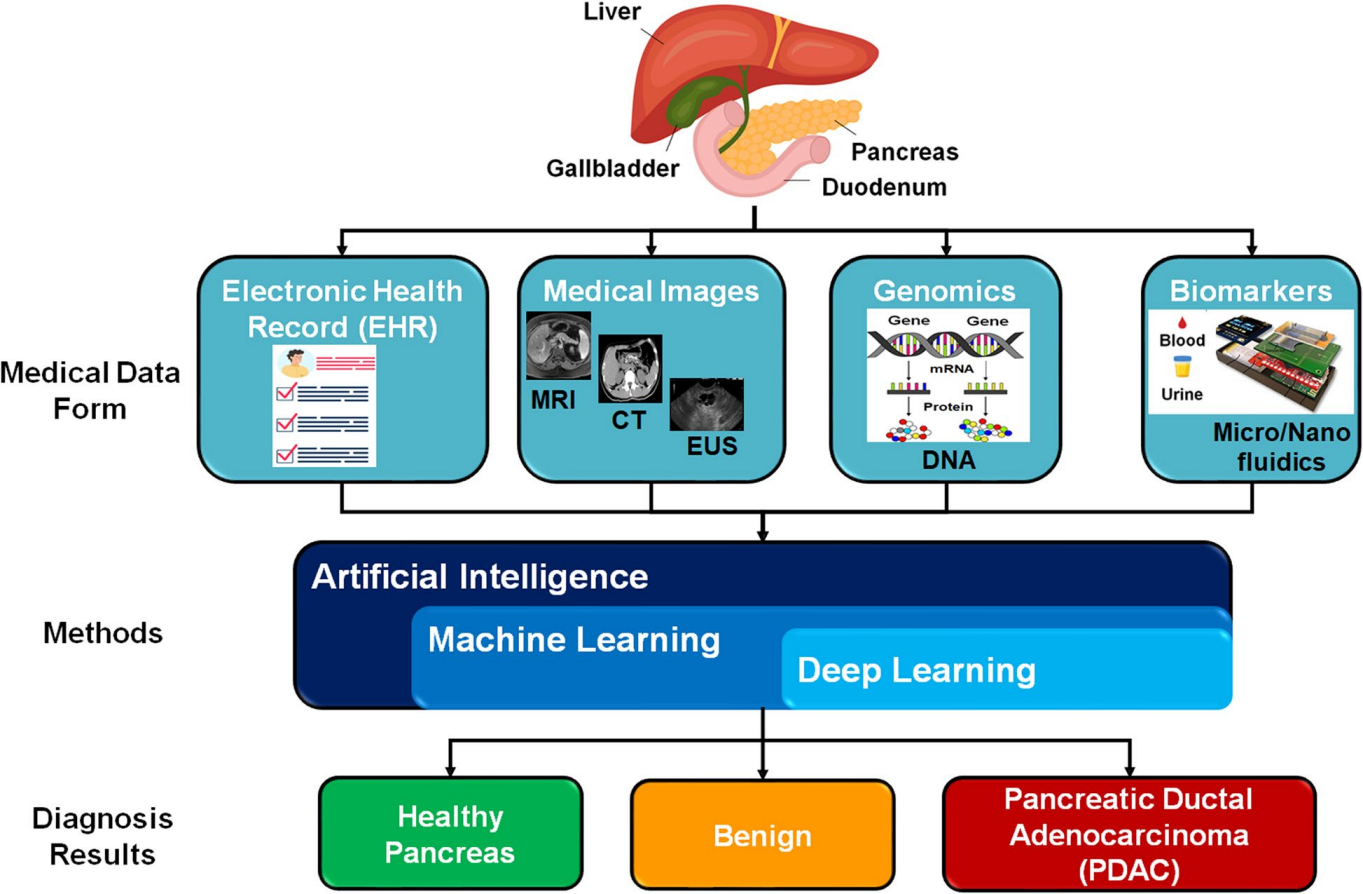
Schematic diagram of applying artificial intelligence techniques to assist diagnosis of pancreatic cancer patients using different forms of medical data

Machine learning (ML) and deep learning (DL) techniques have recently become the core of computer-aided diagnosis (CAD) that can deal with different forms of clinical data, medical images, genomics, and biomarkers. Figure 1 shows a generic schematic diagram of AI-based applications to categorize pancreatic patients into three main groups, namely healthy and two diseased cases of benign and PDAC, based on various forms of input medical data. ML models can learn from patient data in a supervised or unsupervised manner to predict the health status of the pancreas, as proposed in previous studies [22, 27–29]. Advanced DL methods can learn from complex, interrelated, and non-linear features in medical datasets to gain higher diagnostic ability. Hence, some studies employed DL models to detect PDAC tumors using medical imaging modalities, such as multi-parametric MRI [30, 31] and CT [32]. Convolutional neural network (CNN) is one of the main DL architectures for accomplishing medical diagnosis tasks of cancer tumors [33, 34]. Recurrent neural networks (RNNs) are also widely used as a deep learning model for processing sequential data [35]. One of the most common types of RNNs is Long short-term memory (LSTM) networks, which can be integrated with CNNs to improve classification performance in many medical applications [36–38] and PC detection in EUS images [39].

In this article, we propose a new DL model to enhance diagnostic procedures of pancreatic patients using urine biomarkers. This study contributed the following advancements:

- Integrated one-dimensional (1D) CNN with LSTM has been proposed to aid the accurate detection of PDAC based on inexpensive urine biomarkers.
- A comparative evaluation of different ML and DL models has been done to verify the promising results of our developed 1D CNN + LSTM for identifying diseased pancreas cases of benign and PDAC.
- The developed model achieved outperformance in the accurate multi-class classification of pancreatic patients into three groups, namely healthy pancreas, benign, and PDAC cases versus other AI-based models in the current existing state-of-the-art studies.

The rest of this article is structured as follows. Section "Related Works" gives a review of the related works including different clinical modalities with previous ML and DL models to identify PC cases. Section "Dataset and methods" describes both the tested dataset of urine biomarkers and our developed 1D CNN + LSTM classifier in detail. Experiments including results evaluation and discussion of this study are presented in Sects. "Medical data" and "1D Convolutional Neural Network", respectively. At the end of the paper, the conclusion and future directions of this research work are given in Section "Long short‑term memory layer".

## Related Works

This section explores how AI can support early diagnosis of PDAC using different diagnostic methods. We focus on early diagnosis systems based on urine proteomic biomarkers because it is the ultimate goal of this study. A population-based study made by Lee et al*.* [28] represented a predictive model for the early screening of high-risk patients. They accredited that their diagnostic model will support medical care community to know the risk of pancreatic cancer. Their study was built on Taiwan Health Insurance Database (NHIRD). They used four models including logistic regression (LR), deep neural networks DNN, ensemble learning, and voting ensemble to develop their predictive model. The model achieved accuracy ranging from 73 to 75%, and the area under curve (AUC) from 0.71 to 0.76.

Many studies have utilized AI techniques to assist radiologists with interpreting medical images. Liang et al*.* [30] developed a CNN model for auto-segmentation of pancreatic gross tumor volume (GTV) in multiparametric MRI. They employed a square window-based CNN architecture with three convolutional layer blocks for automatic segmentation of the pancreatic GTV. They achieved mean values and standard deviations of the performance metrics on the test set as, dice similarity coefficient (DSC) = 0.73 ± 0.09 and mean surface distance (MSD) = 1.82 ± 0.84 mm. Chen et al*.* [32] validated a new deep learning (DL)–based tool to detect pancreatic cancer on CT scans with reasonable sensitivity for tumors smaller than 2 cm. Their DL tool distinguished between CT malignant and control studies with 89.7% sensitivity, 92.8% specificity, and 0.95 AUC. In addition, the EUS imaging modality needs real-time decision support to differentiate between pancreatic cancer (PC) and non-pancreatic cancer (NPC) lesions. Tian et al. [37] suggested that the YOLOv5m would generate attractive results and allow for real-time detection using EUS images. The suggested model resulted in 95% sensitivity, 75% specificity, and 0.85 AUC.

On the genomic scale, Long et al*.* [27] integrated data mining and multi-omics data for the identification and validation of oncogenic biomarkers of pancreatic cancer. They constructed their prediction model based on a random forest (RF) algorithm because it is an easy-to-comprehend approach. They successfully explored hidden biological insights from multi-omics data and suggested robust biomarkers for early diagnosis, prognosis, and management of PC. The proposed RF model reported an accuracy of 96%.

Using blood samples, Lee et al*.* [13] identified (miRNA) biomarkers derived from blood serum and used them to build the prediction model for PC. They selected 39 miRNA markers using a smoothly clipped absolute deviation-based penalized support vector machine (SVM) and built a PC diagnosis model. Their model obtained an accuracy of 93% and an AUC of 0.98. Hsu et al*.* [14] suggested a new machine-learning model that combines plasma-based biomarker CA19-9 and methylation signals to build a joint multi-omics prediction model for PDAC. This approach achieved a sensitivity of 93% and a specificity of 96%. Ko et al*.* [15] combined machine learning and nanofluidic technology to diagnose PC using exosomes. They developed a multichannel nanofluidic system to analyze crude clinical samples. Then, the linear discriminant analysis (LDA) algorithm is applied to these exosomes to assist in the final diagnosis of cancer patients. This prediction model resulted in an AUC of 0.81 for classifying pancreatic tumors versus healthy samples.

For urine specimens, Debernardi et al*.* [18] identified diagnostic (miRNAs) for early-stage PDAC. They applied LR algorithms to determine the discriminatory candidate miRNA biomarkers. The best results of these models were a sensitivity of 83.3%, a specificity of 96.2%, and an AUC of 0.92. Blyuss et al*.* [40] developed a urine biomarker-based risk (PancRISK) score for stratified screening of pancreatic cancer patients. This model was built based on the three-protein biomarker panel in addition to urine creatinine and age. They compared the results of several ML algorithms including neural network (NN), random forest (RF), support vector machine (SVM), neuro-fuzzy (NF) system, and LR model. Then, they used LR to incorporate it into a PancRISK score. The PancRisk score can stratify between two cases (PDAC) and controls (healthy patients), resulting in a specificity of 90% and AUC of 0.94. ALPU et al*.* [41] studied different regularization methods based on the LR model. This comparative study was conducted on the developed biomarker panel in [19]. It is found that the LR model with adaptive group lasso estimator outperformed other regularization techniques in terms of performance measures. The best classification model resulted in an accuracy score of 76% and an AUC of 0.77. A deep-learning-based PDAC diagnostic system was proposed in [42]. The proposed system used an enhanced CNN model to classify pancreatic diseases based on a multi-categorical urine biomarker panel, achieving 95% accuracy and 0.97 AUC.

## Dataset and methods

### Medical data

The public dataset of this study was collected by Debernardi et al*.* [19]. It includes four featured urinary biomarkers, which are creatinine, LYVE1, REG1B, and TFF1. Creatinine is a protein that indicates the functionality of the kidney. YVLE1 is an acronym for lymphatic vessel endothelial hyaluronan receptor 1. It is a protein that potentially has a role in malignant tumors. The third biomarker REG1B is also a protein and may be associated with regenerating cells of the pancreas. Finally, trefoil factor 1 (TFF1) is a protein, which is potentially a prognostic biomarker associated with the development of PDAC disease. This dataset contains a total of 590 urine samples. It is divided into three patient groups, namely healthy patients (183 samples), benign and PDAC cases of 208 and 199 samples, respectively, as illustrated in Table 1.

**Table 1:** Clinical dataset characteristics of urine samples associated with pancreatic patients in this study

Health status	Total Samples No	Gender (Sample No.)	The age range in years (median value)
Healthy Patients	183	Female (115)	26 – 89 (58)
		Male (68)	30 – 87 (55)
Benign	208	Female (101)	26 – 82 (53)
		Male (107)	29 – 82 (55)
PDAC	199	Female (83)	42 – 88 (68)
		Male (116)	29 – 87 (67)

### 1D Convolutional Neural Network

**Fig. 2:**
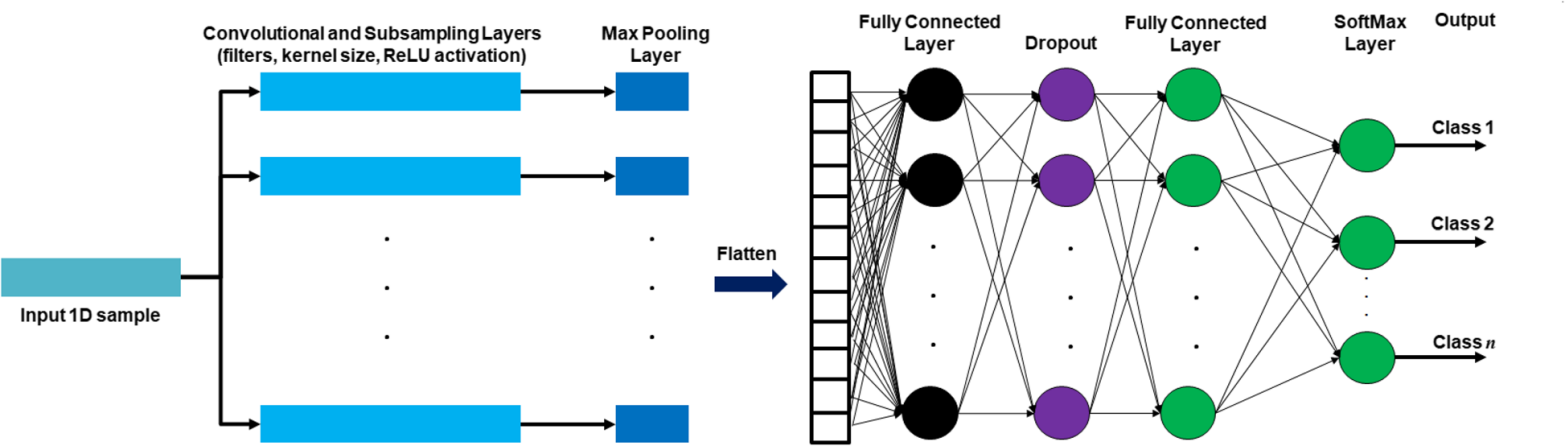
Main layers of 1D convolution neural network for predicting *n* classes

CNN represents an effective tool to extract features and accomplish classification tasks in medicine [33]. In this study, it has been developed to identify pancreas diseases by analyzing 1D data of urine biomarkers. The general architecture of 1D CNN includes convolutional operations, subsampling, dropout regularization, and SoftMax layers [43], as shown in Fig. 2. Each layer of the general 1D CNN architecture can be described as follows. Convolutional and subsampling layers provide feature detection of input 1D samples by performing different filtering operations via convolutions, kernels, and rectifier linear unit (ReLU). The max pooling layer performs a pooling process to select the most prominent features from the overall feature map covered by the predefined filter. The function of Flatten layer is to reshape the multi-dimensional feature map array into a single 1D array, as depicted in Fig. 2. To prevent neural network overfitting, the dropout is applied as a regularization technique for self-modifying the architecture of CNN. Then, the outputs of fully connected network layer are processed by a SoftMax function to give the final output of predicted classes.

### Long short-term memory layer

**Fig. 3:**
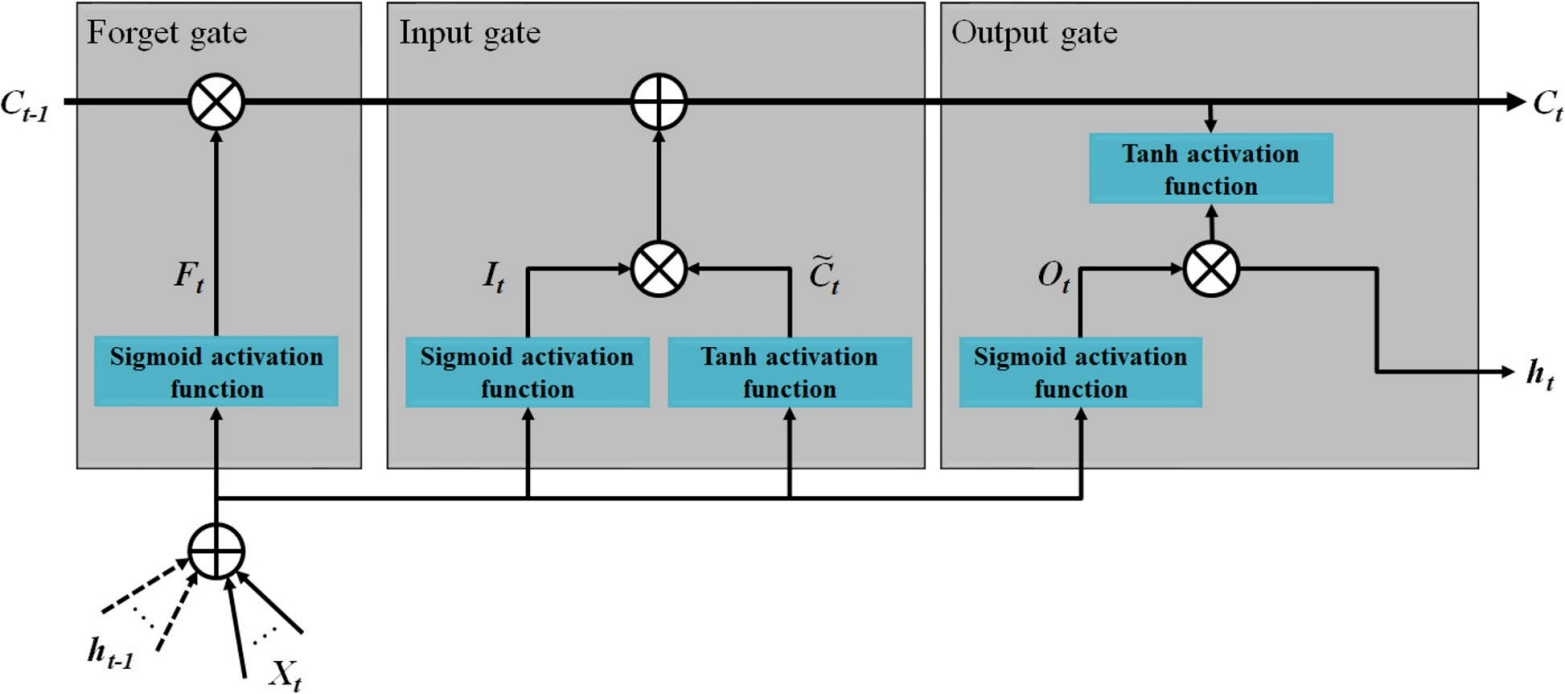
Basic structure of LSTM block

The LSTM is one of the most popular architectures of recurrent neural networks (RNNs) to manipulate data sequentially [44]. The main problem of RNN’s vanishing gradients or long-term dependencies has been solved in the LSTM network, because it can ignore useless information in the neural network for long sequence datasets, such as urine biomarkers in this study. An LSTM layer has mainly three successive gates, i.e., forget gate, input and output gates [44], as shown in Fig. 3. The forget gate is responsible for passing or ignoring data/information flow, as defined by

1 $$F_{\mathit t}\mathit=\sigma\mathit{\left({W_F\times\left[X_t,\;h_{t-1}\right]+b_F}\right)}$$

where *F*_*t*_ is the output of forget gate, *W*_*F*_ and *b*_*F*_ present the weight matrix and bias coefficient associated with forget gate. *X*_*t*_ is the current timestamp input and *h*_*t-1*_ is the previous timestamp hidden state. σ is the sigmoid activation function. In Fig. 3, *C*_*t*_ and *C*_*t-1*_ present the updating and current timestamp cell states, respectively, such that *C*_*t-1* is_ multiplied by *F*_*t*_ as given in (2).

2 $$C_{\mathit t\mathit-\mathit1}\mathit\times F_{\mathit t}\mathit=\mathit{\left\{\begin{array}{l}0,\;F_t=0\\C_{t-1},\;F_t=1\end{array}\right.}$$

The input gate selects the information to be updated using the sigmoid function, *I*_*t*_, then compresses the input sequence in the range of -1 and 1 using the hyperbolic tangent (tanh) function, ${\widetilde{\mathrm{C}}}_{t}$, to add the immediate state to the long-term impact. The mathematical expressions of the input gate are presented as

3 $${\mathit I}_{\mathit t}=\mathit\sigma\left({\mathit W}_{\mathit I}\times\left[{\mathit x}_{\mathit t},{\mathit h}_{\mathit t-1}\right]{\mathit b}_{\mathit I}\right)$$

4 $${\tilde{C}}_{\mathit t}=\tanh\left({\mathit W}_{\mathit c}\times\left[{\mathit X}_{\mathit t},{\mathit h}_{\mathit t-1}\right]+{\mathit b}_{\mathit c}\right)$$

where *W*_*I*_ and *b*_*I*_ present the weight matrix and bias coefficient associated with the input gate, while *W*_*C*_ and *b*_*C*_ are the weight matrix and bias coefficient associated with the candidate state ${\widetilde{\mathrm{C}}}_{t}$.

The third gate of the LSTM block is the output gate, *O*_*t*_, which determines the consideration of the long-term effect and updates the outputs of both the current cell state, *C*_*t*_, and the hidden state, *h*_*t*_, using the sigmoid and tanh functions, as depicted in Fig. 3. The related mathematical expressions of the output gate are given as follows.

5 $$O_{\mathit t}\mathit=\sigma\mathit{\left({W_O\times\left[X_t,\;h_{t-1}\right]+b_O}\right)}$$

6 $$h_{t}=O_{t} \times \tanh \left(C_t\right)$$

where *W*_*O*_ and *b*_*O*_ are the weight matrix and bias coefficient associated with the output gate outcome *O*_*t*_.

### Automated pancreatic cancer classification

**Fig. 4:**
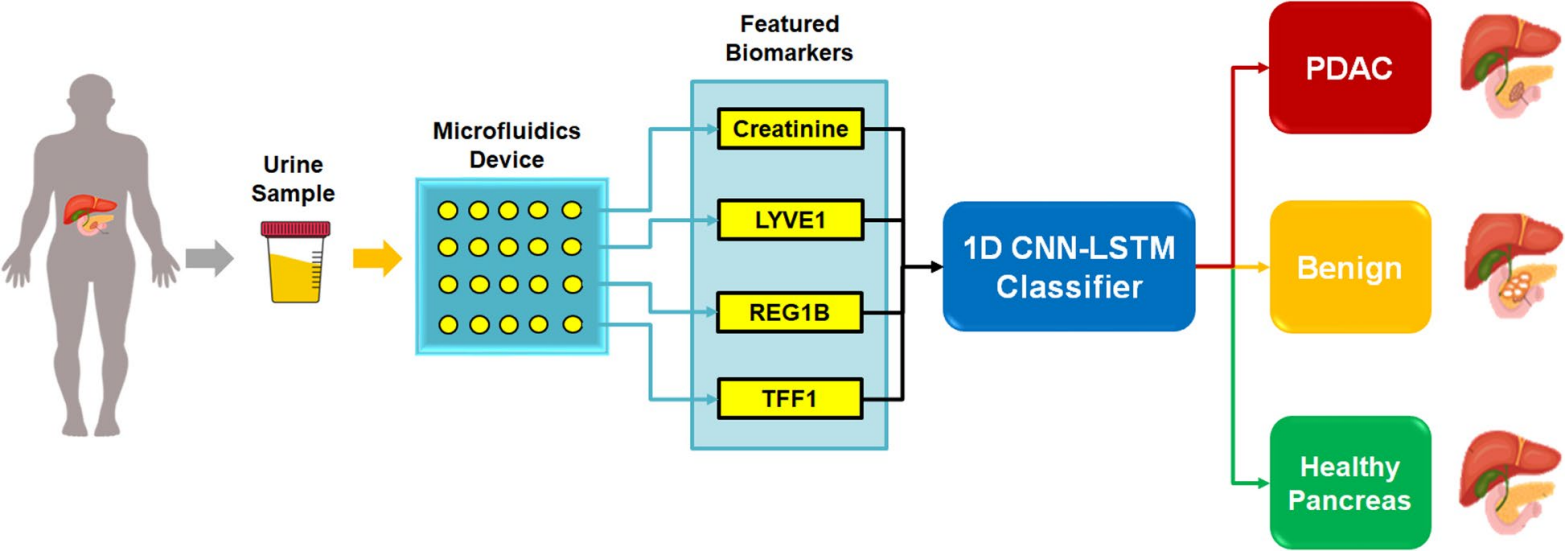
Proposed smart urine biomarkers classification to diagnose pancreatic cancers using 1D CNN-LSTM

**Fig. 5:**
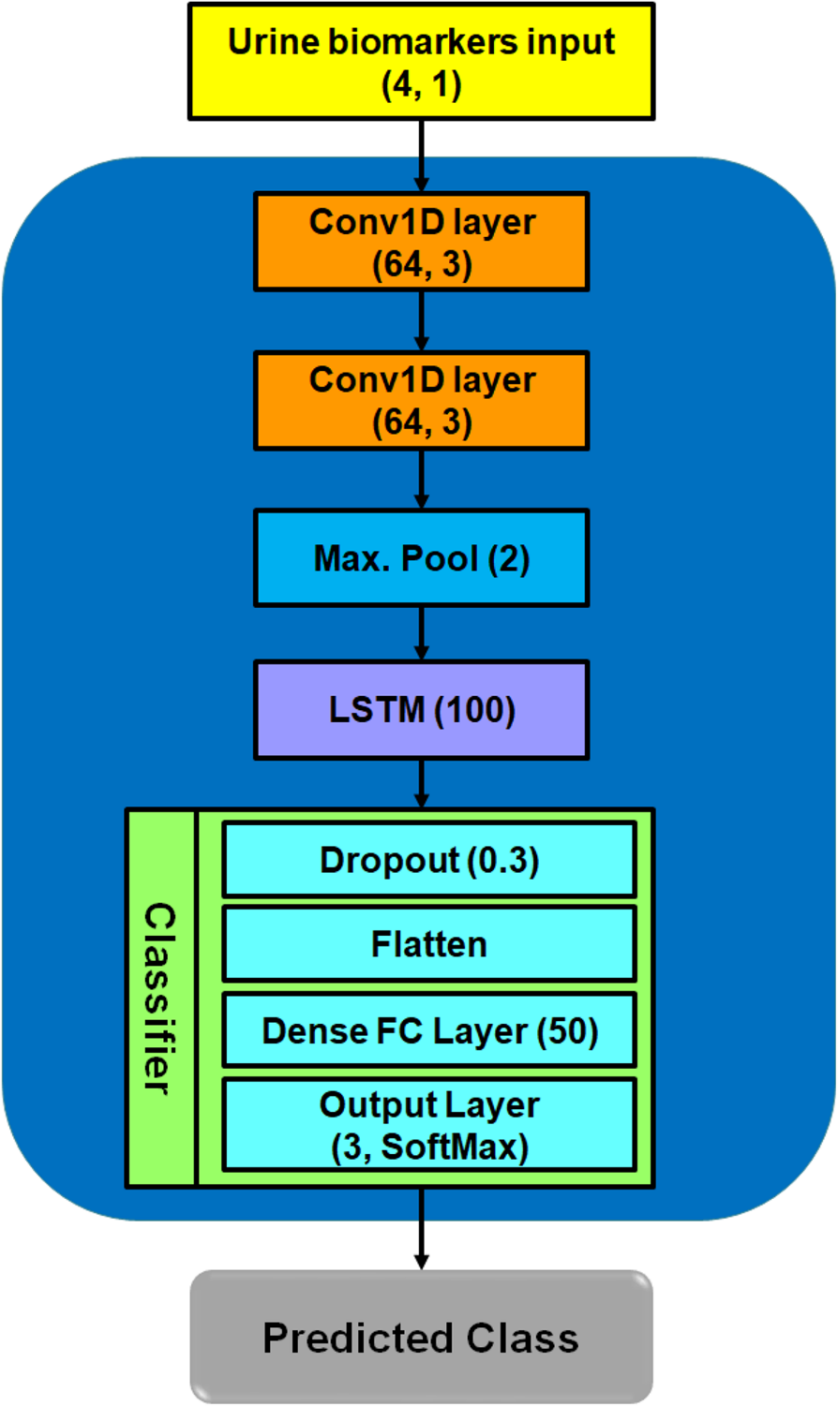
Developed 1D CNN-LSTM model for pancreatic cancer classification

Figure 4 depicts our proposed smart urine biomarkers classification framework for diagnosing pancreatic patients using 1D CNN-LSTM model. First, urine samples are taken from the patient. Second, urine microfluidics device is used to extract four featured biomarkers, i.e., creatinine, LYVE1, REG1B and TFF1, as described above. Then, these four urine biomarkers are fed into our developed 1D CNN-LSTM classifier to predict one of three classes, which are healthy pancreas, benign and PDAC diseases, as shown in Fig. 4. Detailed structural layers of the 1D CNN-LSTM model are depicted in Fig. 5. It includes an input data layer, two 1D convolutional layers, one maximum pooling layer, one LSTM, one fully connected dense layer, and final SoftMax output layer. The 1D CNN-LSTM is considered a lightweight deep neural network with fully trainable parameters of 83,8111.

## Experiments

### Experimental setting

Experiments have been retrospectively conducted to analyze the PDAC classification performance of our developed 1D CNN-LSTM and other machine-learning models, based on the public pancreas dataset of urine biomarkers [19]. The implementation of all tested deep classifiers has been done via Anaconda Navigator V2.3 of Python programming language with Tensorflow-Keras packages and web-based interactive computing notebook (Jupyter V6.4) [45]. These experiments were conducted on a high-performance computing (HPC) laptop equipped with 8 GB NVIDIA GeForce GPU, 16 GB RAM, 256 GB SSD and Intel Core i7-12700H (12th Gen) processor.

**Fig. 6:**
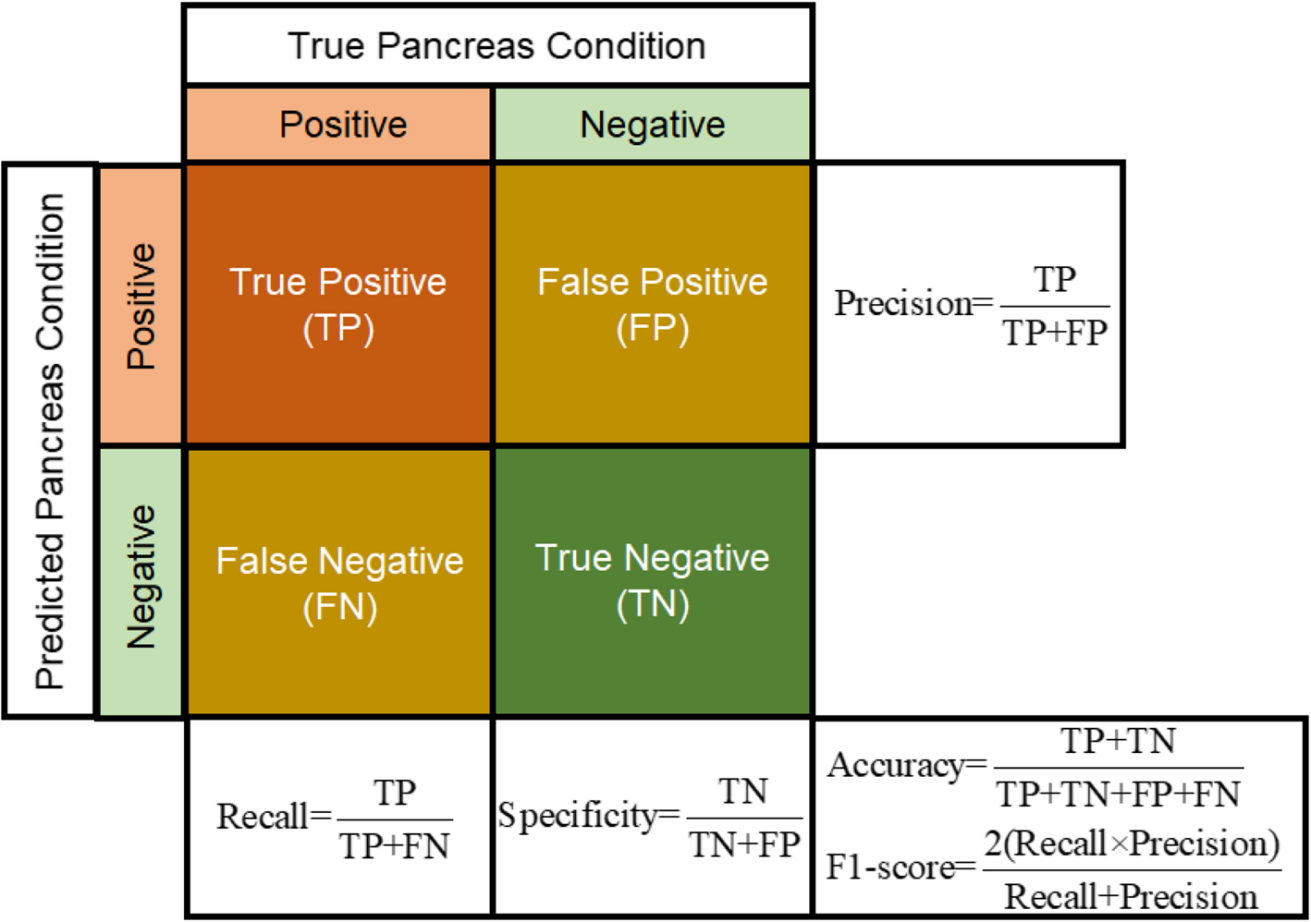
Confusion matrix with evaluation metrics for analyzing the performance of tested classifiers in this study

Using cross-validation estimation [46], a confusion matrix is generated to evaluate the PDAC classification performance of developed 1D CNN-LSTM and other tested models in this study. As shown in Fig. 6, the confusion matrix has four expected outcomes by comparing ground-truth pancreas conditions with the predicted results of any tested classifier. These outcomes are true positive (TP), true negative (TN), false positive (FP), and false negative (FN). In addition, five evaluation metrics, i.e., accuracy, recall or sensitivity, precision, F1-score and AUC have been used to verify the performance of all tested classifiers. Here, other models, i.e., RF, multi-layer perceptron (MLP) neural network, and 1D CNN without LSTM have been implemented to be compared with the performance of our developed 1D CNN-LSTM model.

For starting the training phase of all tested models, the urine samples dataset, as illustrated in Table 1, was randomly split 80 –20 percent, such that the testing phase used 20% of these urine samples, i.e., 118 of 590 samples for accomplishing multi-class classification procedure of healthy pancreas, benign and PDAC cases.

### 4.2 Results and evaluation

**Fig. 7:**
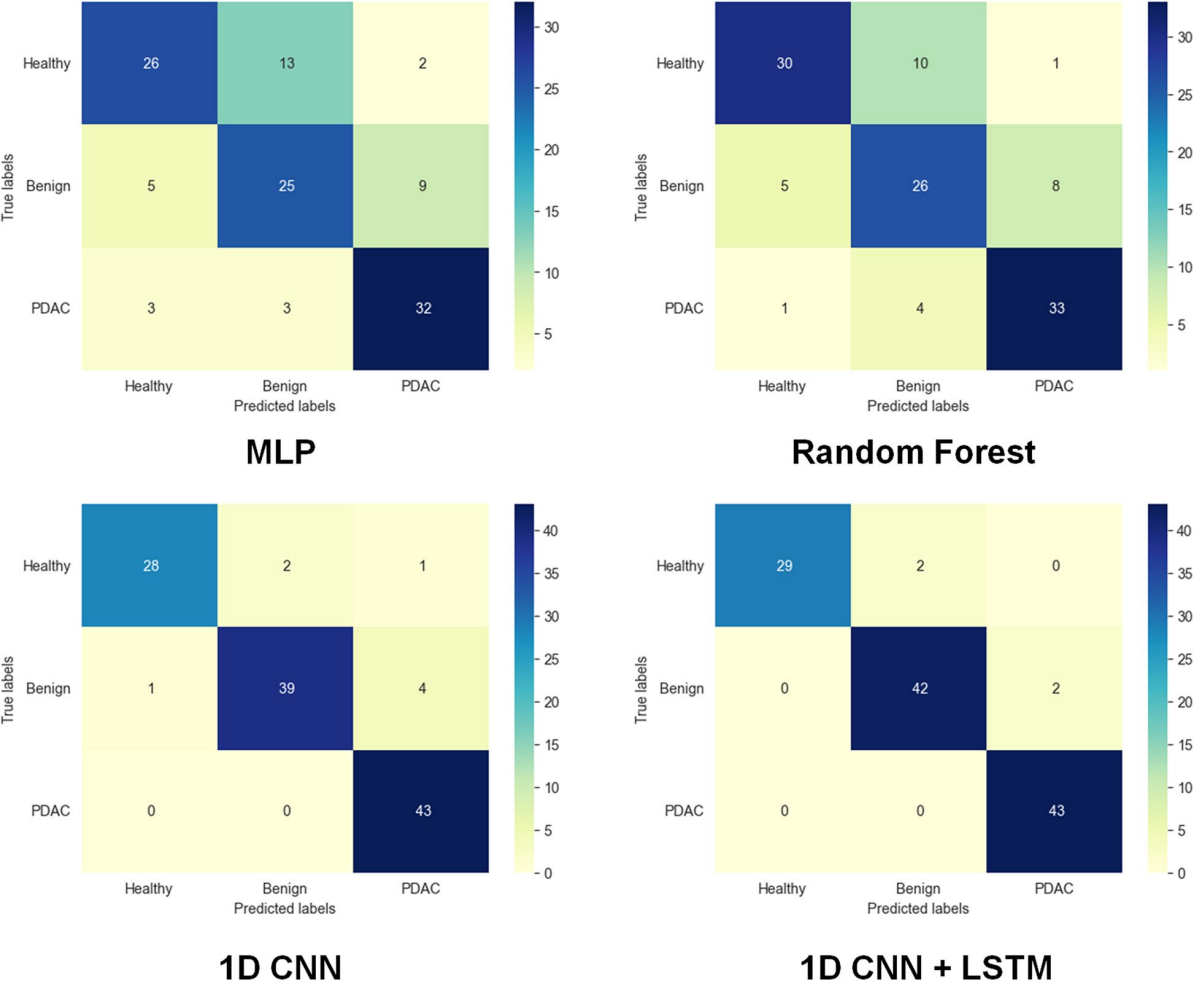
Confusion matrices of the classified healthy pancreas, benign, and PDAC cases using all tested classifiers

Figure 7 shows the confusion matrices for multi-class classification of urine biomarkers into healthy pancreas, benign, and PDAC cases. These results are achieved by our 1D CNN-LSTM and three AI-based models, which are MLP neural network, RF, and 1D CNN. The developed 1D CNN-LSTM model achieved the highest accuracy with no misclassified samples of the PDAC case, but only two urine samples are misclassified for both healthy pancreas and benign cases. In the absence of an LSTM layer, the classification performance of 1D CNN model is decreased, such that the number of misclassified samples is increased for the healthy pancreas (3 samples) and the benign case (5 samples), but no misclassified sample is detected for the PDAC. The MLP neural network and RF could not handle the classification task of urine biomarkers precisely, achieving the worst accuracy scores in these experiments.

Six quantitative metrics, named recall (sensitivity), precision, specificity, F1-score, AUC and accuracy, have been applied to evaluate all tested classifiers, as illustrated in Table 2. The developed 1D CNN-LSTM and 1D CNN still achieved the best accuracy scores of 97% and 93%, respectively. They can be used to diagnose PDAC cases accurately. In contrast, MLP network and RF classifiers achieved the worst accuracy scores of approximately 75%. But the RF model showed better performance than the MLP network model to identify pancreas conditions.

**Table 2:** Evaluation of all tested classifiers to diagnose pancreatic cancers using urine biomarkers

Classifier	Pancreas Condition	Recall	Precision	Specificity	F1-score	AUC	Accuracy
MLP Network	Healthy case	0.63	0.76	0.87	0.69	0.80	0.70
	Benign	0.64	0.61	0.72	0.62	0.69	
	PDAC	0.84	0.74	0.78	0.79	0.79	
Random Forest	Healthy case	0.73	0.83	0.91	0.78	0.89	0.75
	Benign	0.67	0.65	0.77	0.66	0.74	
	PDAC	0.87	0.79	0.84	0.82	0.84	
1D CNN	Healthy case	0.90	0.97	0.99	0.93	**0.99**	0.93
	Benign	0.89	0.95	0.97	0.92	0.95	
	PDAC	**1.00**	0.90	0.93	0.95	0.98	
1D CNN-LSTM	Healthy case	0.94	**1.00**	**1.00**	0.97	**0.99**	**0.97**^**a**^
	Benign	0.95	0.95	0.97	0.95	0.96	
	PDAC	**1.00**	0.96	0.97	**0.98**	**0.99**	

^a^Best performance value is indicated in bold. Abbreviations are already defined in the context

Using the same urine biomarkers analysis, Table 3 illustrates a comparative performance evaluation of our developed 1D CNN-LSTM with other AI-based models in previous studies of automated pancreatic cancer diagnosis. Machine learning models such as logistic regression (LR) [41] could not achieve a high accuracy score (76%) similar to the performance of the RF classifier in Table 2. Additionally, other models like support vector machine (SVM) and neural network (NN) showed an improvement in identifying PDAC cases with AUC = 0.94 [40]. In [42], the application of the employed CNN model achieved 95% accuracy to identify pancreatic cancer conditions. However, our developed 1D CNN-LSTM showed superior classification performance over these previous classifiers by achieving the best values of classification evaluation metrics and the highest accuracy score of 97%.

**Table 3:** Comparative evaluation of developed classifier against the state-of-the-art models to diagnose pancreatic cancers using urine biomarkers

Study	Classifier	Recall	Precision	Specificity	F1-score	AUC	Accuracy
ALPU et al*.* [41]	LR	0.64	0.89	-	0.74	0.77	0.76
Debernardi et al. [19]	LR	0.83	-	96.2	-	0.92	-
Blyuss et al. [40]	NN, RF, SVM, LR	0.81	-	0.90	-	0.94	-
Laxminarayanamma et al. [42]	CNN	**0.96**	0.96	-	0.96	0.97	0.95
Developed 1D CNN-LSTM		**0.96**	**0.97**	**0.98**	**0.97**	**0.98**	**0.97**^**a**^

^a^Best performance value is indicated in bold. Abbreviations are already defined in the context

## Discussion

Intelligent CAD systems have recently become popular in the clinical routine of patients, particularly in the diagnostic procedure of cancer diseases such as the PDAC. Featured urine biomarkers, named creatinine, LYVE1, REG1B, and TFF1 can be extracted from urine microfluidics devices. Here, these four urine biomarkers have been successfully analyzed using our developed 1D CNN-LSTM classifier to identify healthy pancreas, benign and PDAC patients, as depicted in Fig. 4. As illustrated in Tables 2 and 3, the above evaluation results demonstrated that the developed 1D CNN-LSTM outperforms other AI-based models in previous studies with the highest accuracy score of 97%.

Traditional machine learning models, e.g., LR, RF, and SVM showed insufficient performance to identify pancreatic cancer conditions accurately, as introduced previously in [40]. Therefore, supervised 1D CNN-LSTM classifier has been developed to perform automated multi-class classification of 1D urine biomarkers, identifying the health status of pancreatic patients correctly. As described above, the advantageous architecture of the LSTM block showed its capability to ignore useless information in the neural network for long sequence datasets, such as urine biomarkers. Hence, the LSTM layer in our developed model (see Fig. 5) has a main role in significantly improving the classification performance of 1D CNN from an accuracy score of 93% to 97%, as given in Table 2. Moreover, it showed better classification performance than the previous CNN model (95% accuracy) [42], as illustrated in Table 3. Furthermore, the structure of the developed 1D CNN-LSTM model is simple and efficient to achieve targeted diagnostic procedures for pancreatic cancers without high-cost computing resources, e.g., GPUs.

The lack of public medical datasets is a common problem for training supervised learning models, because the number of training samples affects mainly their classification performance. Therefore, accuracy scores of CNN and machine learning classifiers of pancreas cancers are relatively limited to 97%. Consequently, developing deep learning models such as a generative adversarial network (GAN) presents a good solution to handle small medical datasets in semi-supervised or unsupervised learning frameworks [47, 48]. Also, meta-heuristic optimization techniques such as Teaching–Learning-Based Optimization (TLBO) [49, 50] can be applied to automatically update the design of the 1D CNN-LSTM model. Nevertheless, our developed classifier is still capable of achieving a successful and automated diagnosis of pancreas cancer diseases based on urine biomarkers.

## Conclusion and future work

In this article, a new efficient 1D CNN-LSTM model is successfully developed for multi-class classification of pancreas cancer patients using featured urine biomarkers. The classification results categorize the pancreas condition into healthy pancreas, benign and PDAC cases. The developed model achieved the highest values of evaluation metrics including an accuracy of 97% compared to other machine-learning and CNN-based models in the literature, as illustrated in Table 3. Developed CNN models with and/or without the LSTM layer achieved accurate identification of tested PDAC samples, as depicted in Fig. 7.

The main prospect of this research work is to integrate our developed 1D CNN-LSTM with an actual urine microfluidics device for conducting online clinical trials on urine samples of pancreatic cancer patients. Additionally, the Internet of medical things (IoMT) technology can be utilized in this field of study to provide a mobile-based automatic diagnosis of patient samples via medical cloud services.

## Data Availability

The data that supports the findings of this research is publicly available as indicated in the references.

## Abbreviations

PDAC: Pancreatic Ductal Adenocarcinoma
1D CNN: One-Dimensional Convolutional Neural Network
LSTM: Long Short-Term Memory
PC: Pancreatic Cancer
HER: Electronic Health Record
MRI: Magnetic Resonance Imaging
CT: Computed Tomography
EU: Endoscopic Ultrasound
Immuno-PET: Immuno-Positron Emission Tomography
TCGA: The Cancer Genome Atlas
CA19-9: Carbohydrate Antigen 19–9
miRNAs: Circulating micro RNAs
ctDNA: Circulating tumor DNA
CTC: Circulating Tumor Cells
cfDNA: Cell-free DNA (cfDNA),
cfRNAs: Cell-free RNAs
AI: Artificial Intelligence
ML: Machine learning
DL: Deep Learning
CAD: Computer-Aided Diagnosis
RNNs: Recurrent neural networks
LR: Logistic Regression
DNN: Deep Neural Networks
GTV: Gross Tumor Volume
MSD: Mean Surface Distance
NPC: Non-Pancreatic Cancer
RF: Random Forest
SVM: Support Vector Machine
LDA: Linear Discriminant Analysis
NN: Neural Network
NF: Neuro-Fuzzy
YVLE1: Lymphatic Vessel Endothelial hyaluronan receptor 1
TFF1: Trefoil Factor 1
ReLU: Rectifier Linear Unit (ReLU)
HPC: High-Performance Computing (HPC)
TP: True Positive
TN: True Negative
FP: False Positive
FN: False Negative
MLP: Multi-Layer Perceptron (MLP)
GAN: Generative Adversarial Network (GAN)
TLBO: Teaching–Learning-Based Optimization
IoMT: Internet of medical things
